# Update on Cyber Health Psychology: Virtual Reality and Mobile Health Tools in Psychotherapy, Clinical Rehabilitation, and Addiction Treatment

**DOI:** 10.3390/ijerph19063516

**Published:** 2022-03-16

**Authors:** Pasquale Caponnetto, Mirko Casu

**Affiliations:** 1 Department of Educational Sciences, University of Catania, 95123 Catania, Italy; 2 Center for Tobacco Prevention and Treatment, University Hospital “Policlinico G.Rodolico-San Marco”, University of Catania, 95123 Catania, Italy; 3 Department of Clinical and Experimental Medicine, Center of Excellence for the Acceleration of Harm Reduction (CoEHAR), University of Catania, 95123 Catania, Italy

**Keywords:** cyber health psychology, Virtual Reality, mHealth, mCessation, smartphone applications, addiction, smoking cessation, clinical rehabilitation, addiction treatment, psychology

## Abstract

(1) *Background*: we investigated and analyzed the most recent implementations of technology in the fields of psychotherapy, clinical rehabilitation, and addiction treatment. (2) *Methods*: from December 2021 to January 2022, we conducted a review aimed at identifying the recent implementations of technology in cyber health psychology, with particular reference to Virtual Reality in psychotherapy, mHealth tools in clinical rehabilitation, and smartphone applications in the treatment of addiction to substances of abuse, searching for relevant studies in the databases PubMed, Web of Science, Google Scholar, Health & Medical Collection, and APA PsycArticles. (3) *Results*: the tools analyzed are in constant development and are increasingly used, with good results, and further technological progress could lead to even better treatment outcomes; as far as mHealth tools and smartphone applications are concerned, anti-smoking Apps are the most widespread, followed by those for the treatment of alcohol use disorder, and there is no presence of Apps for the treatment of heroin, cocaine, or crack addiction. (4) *Conclusions*: the results of the review indicate that these technological tools are increasingly used and are, in principle, effective and have numerous advantages, including low cost and versatility.

## 1. Introduction

In recent years, there has been more and more talk of cyber health psychology [1] and the implication that new technologies can have in the diagnosis, treatment, and rehabilitation of psychopathological issues in the field of mental health, ranging from post-traumatic stress disorder (PTSD) [2] to addiction to substances of abuse [3,4,5].

Among the most used technological tools is Virtual Reality (VR), an immersive experience of contextual simulation in which the subject wears a head-mounted display (HMD) and orients themselves in a three-dimensional space that has been virtually developed. It is also possible to actively interact with the environment through inputs transmitted with a controller or keyboard or, with the latest Virtual Reality models, through gloves with sensors or motion detection techniques [6]. Virtual Reality is used, for instance, in the treatment of head trauma [7] and Parkinson’s disorder [8]. It has also been used in psychotherapies with patients suffering from schizophrenic spectrum disorder, with promising results [6].

Moving to the side of mobile technologies, the use of Mobile Health (mHealth) is steadily increasing. mHealth is a relatively new area of healthcare; in its 2011 report, the World Health Organization defines it as “the use of mobile and wireless technologies to support the achievement of health goals; it has the potential to transform the face of healthcare provision around the world” [9]. Although mHealth does not have a standard definition, it is considered a medical and public health practice supported by mobile devices, such as cell phones, patient monitoring devices, personal digital assistants, and other wireless devices [9]. The capillarity now achieved by radio infrastructures makes mHealth technologies functional. Mobile connection networks, in 2016, reached 84% of the world population [10]; according to Pew Research Center, 95% of Americans have a generic mobile phone, while 85% use a smartphone [11]. With regard to Italy, 100% of the Italian population is covered by mobile connection networks, both 3G and 4G bands; 92% of the population own a mobile phone [12]. mHealth techniques are frequently used, independently by the subjects or inserted in specific rehabilitation programs, for the treatment of addiction to substances of abuse, such as nicotine, or in interventions aimed at supporting individuals on smoking cessation paths; in these cases, we speak of *mobile phone-based smoking cessation support*, or mCessation interventions [13]. Moreover, mHealth programs based on smartphone applications are used in alcohol use disorder treatments [4] and, albeit to a much lesser extent, in heroin addiction treatments [5].

Henceforth, these topics are explored to provide a clear and schematic summary of materials, methods, and results in order to discuss an overview of the current state of technological evolution and application in the field of cyber health psychology.

## 2. Materials and Methods

### 2.1. Search Strategies

From December 2021 until the date of submission of the present article (January 2022), we searched the databases PubMed, Web of Science, Google Scholar, Health & Medical Collection, and APA PsycArticles for relevant studies using the following search term strings: (“virtual reality therapy application”) OR (“mhealth rehabilitation”) OR (“smartphone app smoke cessation”) OR (“alcohol use disorder smartphone app”) OR (“heroin addiction smartphone app”) OR (“cocaine addiction smartphone app”). The electronic searching was supplemented by hand searching the reference lists in the included review articles to identify any additional sources. This review was fully conducted according to PRISMA guidelines 2020 for systematic reviews [14].

### 2.2. Eligibility Criteria

We included every article meeting the following criteria:

(a) All studies and reviews published in indexed journals and indexed in PubMed, Web of Science, Google Scholar, Health & Medical Collection, and APA PsycArticles.
(b) Studies related to Virtual Reality in psychotherapy, mHealth, mCessation, smartphone applications used in clinical rehabilitation, and addiction treatment.
(c) Published from 2015 until the date of submission of the present article.

## 3. Results

### 3.1. Characteristics of the Included Studies

**Figure 1:**
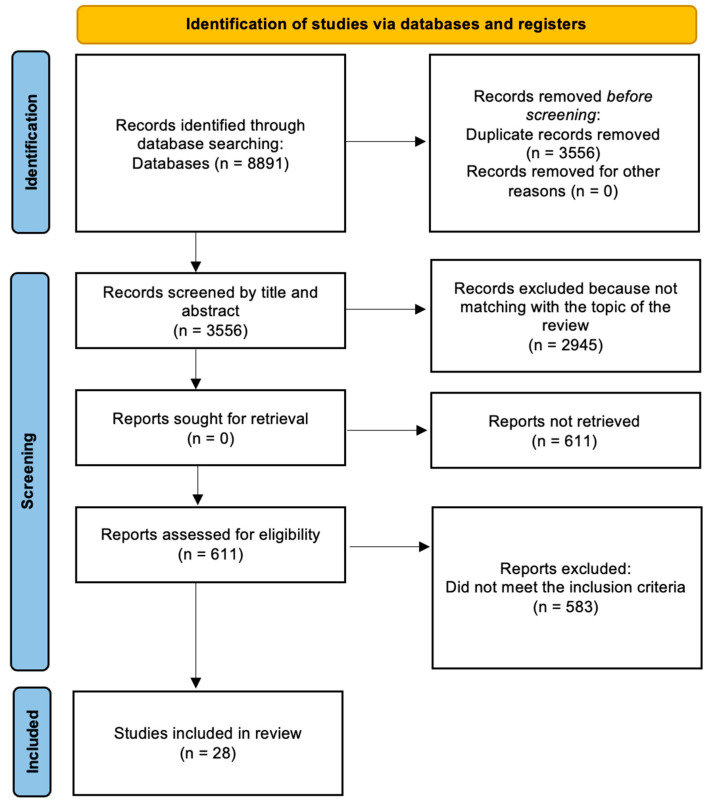
PRISMA (2020) flow diagram.

The database search identified a total of 8891 articles. After excluding duplicates, we found 3556 unique records, which were initially screened, based on title and abstract data. The screening resulted in the selection of 611 articles to be assessed for eligibility criteria; 2945 records were excluded because they did not match the intent of the present review. A total of 611 full-text articles were assessed for eligibility, and 28 out of these met the inclusion criteria and were included in the review; 583 were excluded because they did not meet the inclusion criteria (flow diagram, Figure 1).

### 3.2. The Implementation of Virtual Reality in Psychotherapeutic Treatments

Virtual Reality was found to be an effective tool in mental health fields. In a randomized controlled trial for social anxiety, Bouchard et al. [15] reported that conducting exposure in VR was found to be more effective post-treatment than in vivo on the primary outcome measure and one secondary measure; furthermore, improvements were maintained at the 6-month follow-up, suggesting that using VR can be advantageous over standard cognitive–behavioral therapy (CBT) as a potential solution for treatment avoidance and as an efficient, cost-effective, and practical medium of exposure.

This tool has also been used in therapies with patients suffering from schizophrenic spectrum disorder with promising results, proving to be particularly effective in the treatment of psychotic symptoms, such as delusions and hallucinations, and cognitive and social skills; there is a general agreement in the literature on the safe, tolerable, and long-term persistence of the therapeutic effects obtained by immersive VR. In addition, no serious side effects have been reported [6]. The usefulness of Virtual Reality in the treatment of psychotic disorders derives from the fact that this tool can represent social environments that trigger responses, reactions, and emotions equivalent to what the real world in a given context would create in the mind of patients [16,17]; similarly, a virtual person (avatar) will elicit reactions similar to those provoked in real life [18].

Dellazizzo et al. [2] found that treatments employing VR stress-provoking scenarios significantly decreased the frequency and intensity of anger, as well as significantly decreasing core PTSD symptoms; they also analyzed a cognitive training program with immersive VR based on a virtual classroom and electroencephalogram (EEG) biofeedback for symptoms of attention-deficit hyperactivity disorder (ADHD). The program did not show significant improvements in both the VR and non-VR group scores, although the VR group improved the most.

The most recent literature also states that Virtual Reality Exposure Therapy (VRET) proved to be a valid alternative to “In Vivo” therapies (iVET) for the treatment of social anxiety disorder and its various forms. Caponnetto et al. [19] found, through the analysis of various studies, that the effectiveness of these two types of treatment is comparable, stating that “the superiority of VRET over iVET should not be seen as much in the perspective of the reduction in symptoms, since they seem to be equally effective, but in the drastic reduction in the costs to carry out the therapy and in the flexibility that allows the clinician to control all the variables at stake”. The cost effectiveness of VRET may today represent the turning point for wider access to psychological care to currently excluded socioeconomic classes (Table 1).

### 3.3. The Advent of Mobile Health

Mobile Health exploits the use of mobile devices and their functionalities, and it includes simple strategies, such as the use of the short messaging service (SMS or text messages), and more complex strategies, such as using smartphone applications (Apps), telecommunication systems 3G/4G, general packet radio service (GPRS), global positioning system (GPS), and Bluetooth technologies [9]. More recently, the use of sensors to monitor and provide feedback to suppliers and patients about biological parameters is fueling new areas of both research and development. The fact that it is such a widespread tool makes the smartphone also extremely useful for clinical rehabilitation.

#### 3.3.1. mHealth and Clinical Rehabilitation

Choi et al. [20] demonstrated the effectiveness of stroke rehabilitation programs using video games in Virtual Reality on smartphones. The mobile game-based VR program effectively promoted upper extremity recovery in patients with stroke; in addition, patients completed the treatment without adverse effects and were generally satisfied with the program. As stated by the authors, “this mobile game-based VR upper extremity rehabilitation program can substitute for some parts of the conventional therapy that are delivered one-on-one by an occupational therapist. This time-efficient, easy to implement, and clinically effective program would be a good candidate tool for telerehabilitation for upper extremity recovery in patients with stroke”. We can see that one of the recurring positive factors of new technologies, in addition to easy applicability, is the low cost of use.

Song et al. [21] used smartphones as telemonitoring platforms for rehabilitation exercises with patients with coronary heart disease; the results of this study showed that patients in the intervention group had more significant improvement in exercise tolerance compared with those in the control group. The authors stated, however, that “it must be noted that both study arms indicated a tendency towards improvement regardless of intervention; although, the improvement was more obvious in group A”. Improvements in exercise tolerance can lead to greater consistency in exercise rehabilitation programs, producing better outcomes and increasing patients’ compliance.

Nehrujee et al. [22] worked on successfully verifying the usability of a smartphone-based gaming system for vestibular rehabilitation. Unlike all existing systems for vestibular rehabilitation, VEGAS—the smartphone-based gaming system VR HMD used in the study—is convenient, compact, portable, and suitable for training a wide range of exercises both in clinics and in patients’ homes. According to the authors, the system was reported to be highly usable by both healthy subjects and patients included in the study; furthermore, they “strongly believe that the smartphone-based HMD approach has significant potential to become an affordable, portable, and safe tool for vestibular rehabilitation”, therefore remarking how these instruments, which improve year after year, are functional and convenient from every point of view (Table 2).

#### 3.3.2. Mobile Phone-Based Smoking Cessation Support (mCessation)

mHealth systems were also used as part of interventions aimed at supporting individuals on smoking cessation paths; in these cases, as already written in the Introduction, we speak of *mobile phone-based smoking cessation support*, or mCessation interventions [13]. According to the data, in 2019, there were over 1 billion smokers worldwide, and they consumed over 7 trillion cigarettes. The proportion of people who smoke globally has decreased since 1990, while, with population growth, the absolute number of smokers has grown in many parts of the world; moreover, also since 1990, the global prevalence of smoking among subjects aged between 15 and 24 years has decreased significantly, although the absolute number of young smokers has increased in some regions due to the growth of the population itself. In 2019 alone, tobacco smoking was held directly responsible for the deaths of 7.69 million people [23]. These and previous data have pushed toward the search for even better systems to combat the spread of tobacco and smoking addiction, identifying a useful ally first in the mobile phone and then in the smartphone.

A review conducted by Whittaker et al. [13], based on 26 studies with a total of over 33,000 participants, highlighted how the use of mCessation, in particular, the use of automated text message systems delivered to mobile phones, led to higher termination rates than the minimum standard for smoking cessation support. The authors specifically argue that there is moderate certainty of the benefit of interventions involving automated text messaging if used in addition to other smoking cessation support compared to only minimal smoking cessation support.

Deutsch et al. [24] described a similar system called *Test My Quit* (TMQ), also based on sending text messages on mobile phones, and highlighted its potential effectiveness. The design of this system consisted of a tailored smoking cessation intervention delivered 100% through text messaging to an intervention group in comparison with non-smoking-related text messages sent to a control group. The authors concluded the description of the design by asserting that, in addition to specificity and technicality, one of the strengths of *Test My Quit* is its total accessibility on the market, as it is based on text messages.

Liao et al. [25] conducted a text message-based smoking cessation intervention on the Chinese population, called *Happy Quit*, and found positive effects on a small number of subjects in the sample, suggesting greater efficacy on larger samples. This type of intervention was also studied with representative samples of minority populations, such as Somali nationals professing the Islamic religion by Pratt et al. [26] and pregnant women by Forinash et al. [27]; in the first case, positive prospects were recorded especially during the Ramadan period, while, in the second case, the impact of the intervention was minimal (also due to some problems that occurred during the recruitment phase and high drop-out rates).

### 3.4. Smartphone Applications Designed for Smoking Cessation

Another type of mHealth, used in support interventions for smoking cessation, concerns the use of smartphone applications specially designed for this purpose.

Regmi et al. [28] conducted a review involving eight studies of the clinical trial type or interventions, concerning smartphone applications designed for smoking cessation. From the reported results, it is clear that this is an area that is still embryonic but of certain interest and that requires constant further study; the use of such applications appears to produce an increase in cessation rates among smokers, although adherence to the internal functionalities of the application appears to influence cessation rates; “audiovisual features followed by a *quit plan*, progress tracking and sharing features are the most liked and used application features”, although “inconsistency in their association with abstinence or termination rate has been observed”. The authors also report a reduction in relapses attributed to debates carried out on social media among those who quit smoking. Regmi and colleagues conclude by suggesting carrying out studies with applications of this type on larger samples to measure the effectiveness of the intervention more clearly.

Pbert et al. [29] compared the effectiveness of interventions aimed at smoking cessation: two interventions based on smartphone applications specifically designed to help people quit smoking and based on *mindfulness training*, one called *Craving to Quit* (C2Q) and the other *QuitSTART*, while the third intervention involved the use of written material. The selected sample included 146 adolescents. The authors found that “cotinine-validated abstinence (tobacco alkaloid and nicotine metabolite) was similar between intervention conditions and tended to increase with (subject’s) greater engagement in each condition”. They go on to state that “a greater involvement of the C2Q App among heavy smokers was associated with a significantly greater decline in the number of cigarettes smoked, compared to other conditions”. *The Craving to Quit* App was also employed in a randomized controlled experiment conducted by Garrison et al. [30]; although the results led to the conclusion that the *mindfulness training* through the App “did not lead to a reduction in *smoking* rates compared to the control group”, useful data were found to hypothesize that this system may loosen the association between *craving* and smoking, which would be particularly useful in supporting cessation in long-term programs.

Another application studied was *Quit2Heal*, in comparison with the *QuitGuide* of the US National Cancer Institute, by Bricker et al. [31]. In this case, the sample was composed exclusively of cancer patients, and the application showed “promising acceptability and efficacy in helping cancer patients to quit smoking”.

### 3.5. Smartphone Applications Designed for Alcohol Use Disorder

Nicotine addiction is not the only one to be treated with the help of smartphone applications. It is possible to find a fair number of Apps (*n* = 40) designed to help reduce substance use on both Apple’s and Google’s digital stores [32]. Most of the Apps in this category are directed toward the use of alcohol to facilitate abstinence or reduce the drinking amount. Examples of Apps targeting alcohol use are *Saying When*, developed by The Canadian Center for Addiction and Mental Health, and *Sober Grid*, developed by a team of academics and developers to offer a global newsfeed of shared posts on experiences in and insights into recovery and an instant help feature. However, Tofighi et al. stated, after a deep analysis, that the Apps “lacked any clinical evidence of efficacy, there was also no evidence of sustained use”. For instance, “apps claiming to offer peer support via forums or SMS text messaging contact with other users were not active or unresponsive”.

However, a pilot study conducted by Gonzalez and Dulin in 2015 [4] demonstrated the significant efficacy of an intervention for alcohol use disorder with the use of a smartphone App. As suggested by Tofighi and colleagues [32], this difference in the results probably derives from the fact that truly effective Apps are not available on public App stores but rather are bound to invitations provided by a licensed addiction treatment provider.

A longitudinal study performed by Laurens et al. [33] also demonstrated the effectiveness of a smartphone App in treating alcohol use disorder. In particular, the App was called Breindebaas, and the results state that subjects registered a significant reduction in alcohol consumption after 3 weeks and another reduction at 3 months. Positive feedback was also provided regarding the fast-working, simple, and user-friendly design of the App. Almost half of the subjects also reported gaining more control over their alcohol use. However, Breindebaas, the App used, responds to the criticism of Tofighi and colleagues; it is not available on public stores but only on request to developers. This places a limit on the use of effective Apps in this area, thus requiring the subject to contact specialists or treatment programs to gain access to these resources as opposed to what has been seen for Apps designed for smoking cessation.

### 3.6. Smartphone Applications Designed for Heroin Addiction Treatment

There are currently no documented studies involving the use of Apps specifically designed for the treatment of heroin addiction.

We found only one article containing the searched keywords; however, it is an analysis conducted by Schulte et al. in 2016 [5] that investigated the ideal prospects for the development and eventual adoption of a smartphone application in the context of heroin addiction recovery support. It was found that there was a general line of acceptance of the possible use of this tool, although there were some differences on a social and technological level. In particular, there was a greater concern in subjects from China and Taiwan regarding these areas rather than in patients from the USA. This was motivated by the fact that the spread of the smartphone was wider in the United States compared to the two countries mentioned above.

Finally, Tofighi et al. [32] found fewer than 10 (*n* = 6) applications on App digital stores specifically indicated for opioid and heroin addiction. However, most of these Apps have been designed to connect users with peers and not for direct treatment purposes. They indeed claim to provide users access to sober peers via intra-app messaging, help icons, and forums.

### 3.7. Smartphone Applications Designed for Cocaine and/or Crack Addiction

No studies or reviews were found in the literature regarding the use of smartphone applications designed for the treatment of cocaine or crack addiction. However, Tofighi and colleagues [32], in their analysis, did not find any application on digital stores specifically indicated for addiction to cocaine or crack (Table 3). The reasons remain to be understood, which we discuss in the sections to come.

## 4. Discussion

As we saw during this review, technology evolves year after year, and there are sectors, such as cyber health psychology, in which it takes on an increasingly important role [1]. The evolution of Virtual Reality goes hand in hand with the advancement of graphic processing and the increase in the complexity of the virtual environments that can be created, thus producing an even greater immersion of patients and even better results, managing to reduce the limit between the real and the virtual. The immersion produced using Virtual Reality tools is linked to the use of an avatar. In the field of e-health mental interventions, an avatar is a digital self-representation of the person, in line with the identity and character attributed. Through the avatar, the therapist can interact with the patient and the patient can act within the virtual space, simulating behaviors and attitudes that they would display in vivo [34]. The use of avatars in e-mental health interventions represents a nascent area of inquiry. As demonstrated by Rehm et al. [35], the psychotherapeutic applications of avatars are numerous, and avatars seem to serve several functions conducive to the commitment of treatment.

The best potential outcomes of psychotherapeutic treatments involving the use of VR due to graphic advancement are cited by the same authors [19]. The broad spectrum of use of VR is certainly interesting, ranging from the treatment of social anxiety [15] to the treatment of psychotic symptoms in patients with schizophrenic spectrum disorder [6], obtaining equally significant or even more effective results post-treatment [15]. The strengths of this tool, as we have seen, are its low cost and practicality [19], factors that make it easily available. Further determinants that could lead to the use of Virtual Reality, according to the results by Bisso et al. [19], are the following: first, it allows for effective and short interventions without the use of drugs and, therefore, pharmacological side effects; secondly, it is highly customizable to the needs of the individual patient; finally, in some cases, it also allows the treatment of psychotic symptoms of drug resistance. For even greater effectiveness and better treatment outcomes, it is always recommended to combine the tool with a parallel psychotherapy path or, in any case, integrate the tool and implement a multi-faceted approach [19].

The advent of mHealth tools, in direct correlation with the widespread diffusion of telecommunication tools, has certainly marked a great step forward regarding the possibility of expanding the range of useful tools in the medical and mental health fields. The whole literature agrees in affirming the effectiveness of mobility-based tools, whether it is intervention programs based on the transmission of SMS [13,23,24,26,27] or alternative uses of the smartphone, as in the case of remote telemonitoring [21]. The charm of these instruments is that we often come across contaminations, as in the case of mHealth systems that involve the use of smartphones but which are based on Virtual Reality equipment and HMDs [20,22].

The use of mHealth technologies is also very interesting concerning the treatment of addiction to substances of abuse, in particular, nicotine, alcohol, heroin, cocaine, and crack.

The treatment of nicotine addiction, carried out and explored in smoking cessation support programs, is implemented in different ways. The use of mCessation techniques follows the basic use of sending text messages to subjects, thus depriving them of the active and interactive component of the use of a smartphone application [13,23,24,26,27]. Although the interventions that involve textual messages were found to be effective, significantly or moderately, we believe that greater involvement in the treatment is important for the patient. Smartphone applications could therefore be suitable in this regard. The reasons that determine the use of smartphone App-based treatments are their relatively low cost, ease of use, and availability. Compared to in-person treatments, smartphone App-based treatments are more accessible; are cheaper; reduce stigma; improve monitoring; improve standardization; are customizable and scalable; provide direct access to self-management tools and real-time interactive and social support; and place treatment into users’ hands, in context and potentially just in time [30].

The revised literature gives us an idea of the current state of the art in the field of drug addiction treatment using smartphone applications, always remembering that this is an extremely new and embryonic field of research and application. The popularity and the spread of tobacco use have probably contributed to the development of more Apps to support smoking cessation than treating addiction to other substances. Furthermore, this has probably led to a greater diffusion of Apps with this design and, consequently, more feedback and better experimental results. The anti-smoking Apps have the greatest proven effects, as well as different ways of being designed (for example, we have seen Apps based on simple smoking monitoring [28,29] as opposed to Apps based on mindfulness training [30]).

This is different regarding the Apps targeting the use of alcohol; they are difficult to find on digital stores, and, above all, those that are there are less effective and potentially useless [32]. This changes if we consider Apps designed by academics and licensed by treatment providers, limiting any attempt of individual self-administration and binding the patient to an intervention program. In general, however, the Apps given by addiction treatment providers are effective (although there are very few studies in the literature on this subject) [4,33].

Interestingly, at the time of writing this review, there were no smartphone applications aimed at the treatment or use of drugs of abuse, such as heroin, cocaine, and crack. Despite a 2016 preliminary investigation by Schulte and colleagues [5], to date, no applications have been developed in this direction. Tofighi and colleagues [32] report the existence of *n* = 6 applications available in public digital stores indexed for the treatment of opioid or heroin addiction, but it turned out that they are all based on sober peer comparison systems and do not provide any type of path or specific treatment, thus being reduced to virtual social “corners”. As for cocaine addiction, the number of applications was null, and nothing related was found in the literature.

Our hypothesis behind these data is that research is probably proceeding step by step in the development and exploration of these smartphone applications, trying to find a sufficiently effective design and model in smoking cessation to then be able to re-propose the same model for the treatment of other addictions and substances of abuse.

The fact that the only existing applications focus on nicotine and alcohol is probably dictated by the widespread use and abuse of these substances, thus making it a priority to finalize effective treatment models for these substances first and then to invest resources and time for the treatment of heroin and cocaine addiction, which are equally important but certainly less widespread.

Given the exponential growth in the use of mobile Apps to promote health and well-being in recent years, one major limitation is the need to discern quality in mobile Apps. As the technological capabilities behind these Apps increase, so do the potential risks [36]. This challenge is partly due to the lack of a transparent and standardized approach to validation. Several tools address this problem. For example, the Mobile App Rating Scale (MARS) is a well-known standardized tool developed by the Queensland University of Technology by which healthcare Apps can be compared. It is designed to evaluate Apps on criteria of engagement, functionality, aesthetics, and information quality [37]. As noted by Sedhom et al., widely cited generalized mobile App rating frameworks, such as MARS, are nonetheless underdeveloped in clinical appropriateness. The App Quality Assessment Tool or Health-Related Apps (AQUA) [38] combines MARS with Enlight [39], an assessment tool that incorporates therapeutic concepts. However, as with its component frameworks, this tool does not include key technical elements other than data privacy and security. The THESIS approach [40] uses the domains of transparency, health content, technical content, security/privacy, usability, and subjective evaluations. Nonetheless, it does not incorporate clinical evidence.

While these frameworks have an advanced quality assessment, a pragmatic quality assessment tool is still required that incorporates end-user requirements, as well as technical, usability, and clinical dimensions [36].

## 5. Conclusions

In conclusion, with this review, we were able to analyze the current situation and the latest data in the literature regarding Virtual Reality in psychotherapy, mHealth in general, and specifically in clinical rehabilitation and smartphone applications. It is a new and constantly evolving research and application territory, and we cannot wait to be able to use these technological tools increasingly in the context of cyber health psychology, especially after having seen all the benefits and advantages that these tools bring to both therapist and patient.

## Figures and Tables

**Table 1:** Characteristics of Virtual Reality concerning the effects found in psychotherapy treatments and the advantages of this tool.

Characteristics of Virtual Reality	
Effects in Psychotherapy	More effective at post-treatment than in vivo Improvements maintained for at least 6 months Effective in the treatment of psychotic symptoms Decreases the frequency and intensity of anger Decreases core PTSD symptoms Potential benefits in impulsivity treatments
Medium Advantages	Efficient Cost effective Practical Safe Tolerable No serious side effects Flexible, possibility of controlling all the variables at stake

**Table 2:** Forms of mHealth, areas of use in clinical rehabilitation, and medium advantages.

Characteristics of mHealth	
Forms of mHealth tools	SMS (short text messages) Smartphone applications Smartphones as telemonitors Mobile game-based VR systems
Areas of use in clinical rehab	Upper extremity recovery in patients with stroke Rehab exercises with patients with coronary heart disease Vestibular rehabilitation
Medium advantages	No adverse effects General patients’ satisfaction Low cost Time efficient Easy to implement Clinically effective Highly usable Safe

**Table 3:** Summary with the substances of abuse treated and the characteristics of the Apps analyzed.

Substance of Abuse	App Availability	App Effectiveness	App Limits
Nicotine	High availability	Increase in cessation rates Adherence to the internal functionalities appears to influence cessation rates Involvement is associated with decline in the number of cigarettes smoked Promising acceptability and efficacy in helping cancer patients to quit	Inconsistency of appreciation of certain features of the App in association with abstinence or termination rate Mindfulness training through the App did not lead to a reduction in smoking rates
Alcohol	Medium–ow availability	Significant efficacy of an intervention for alcohol use disorder More control gained over alcohol use	Apps on public digital stores lack any clinical evidence of efficacy Apps claiming to offer peer support via forums or SMS text messaging contact with other users were not active or unresponsive Most effective Apps not available on public stores but only on request to developers
Heroin	No availability	No data	No data
Cocaine/crack	No availability	No data	No data

## Data Availability

Not applicable.
